# Supplementary material for: Hybrid Gold Nanorod-Based Nanoplatform with Chemo and Photothermal Activities for Bimodal Cancer Therapy
Source: Int J Mol Sci. 2022 Oct 28;23(21):13109. doi: 10.3390/ijms232113109 (PMC9659131; doi:10.3390/ijms232113109)
Supplement: Supplementary file 1 [file ijms-23-13109-s001.zip › ijms-1953660-supplementary.pdf]

SUPPLEMENTARY MATERIALS FOR:

Hybrid gold nanorod-based nanoplatform with chemo and photothermal activities for bimodal cancer therapy

Lilia Arellano [1, ∝], Eva Villar-Alvarez [1,4,∝], Alejandro Varela [1], Valeria Figueroa [1,2] Javier Fernández [1], Adriana Cambón [1*], Gerardo Prieto [3], Silvia Barbosa [1*], Pablo Taboada [1].

[1] Grupo de Física de Coloides y Polímeros, Departamento de Física de Partículas; Facultad de Física; Instituto de Materiales (IMATUS) e Instituto de Investigaciones Sanitarias (IDIS), Universidad de Santiago de Compostela, 15782-Santiago de Compostela, Spain.
[2] Departamento de Ingeniería Química, CUCEI, Universidad de Guadalajara, Guadalajara. Mexico.
[3] Grupo de Biofísica e Interfases, Departamento de Física Aplicada, Facultad de Física; Instituto de Materiales (IMATUS), Universidad de Santiago de Compostela, 15782-Santiago de Compostela, Spain
4 Nanostrcutured Funtional Group, Catalonian Institute of Nanotechnology (ICN2), Universidad Autónoma de Barcelona Campus, Av. Serragalliners s/n, 08193 Bellaterra, Barcelona.

* Correspondence: adriana.cambon.freire@usc.es; silvia.barbosa@usc.es
∝ These authors contribute equally to this work.

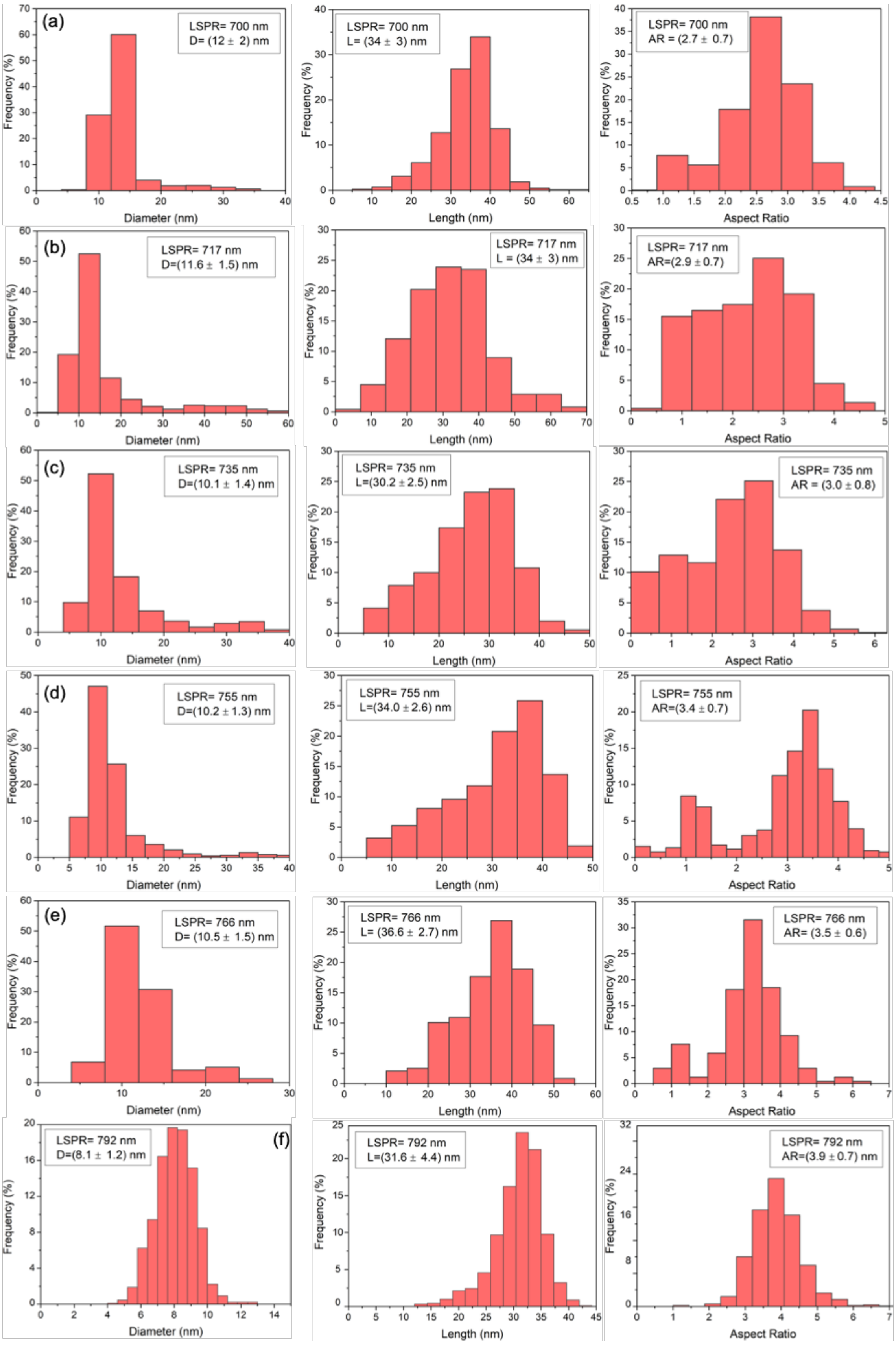

Figure S1: Length, width and AR frequency distributions (from left to right) derived from TEM measurements for several types of obtained AuNRs. Each row displays a longitudinal LSPR band centered at (a) 700 nm, (b) 717 nm, (c) 735 nm, (d) 755 nm, (e) 766 nm, and (f) 792 nm (N = 800). Measurements were made using the ImageJ software.

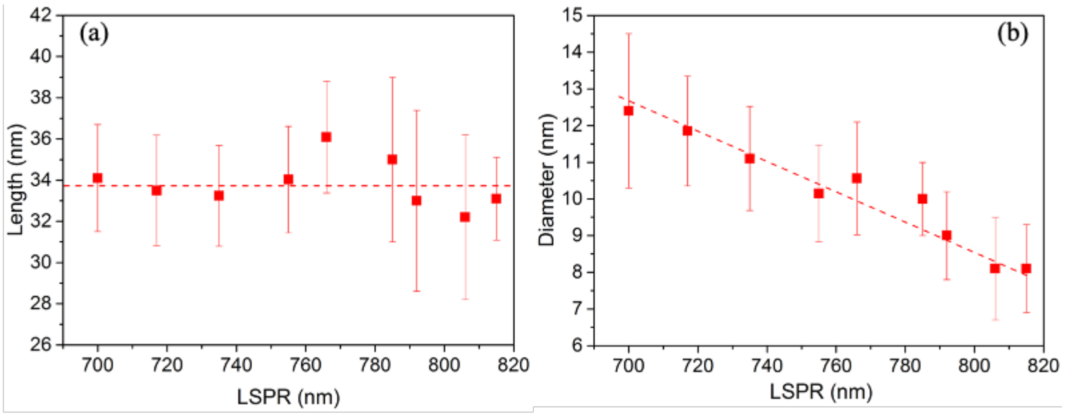

Figure S2: Changes in (a) AuNR length, and (b) width upon changes in $AgNO_3$ concentration during particle synthesis.

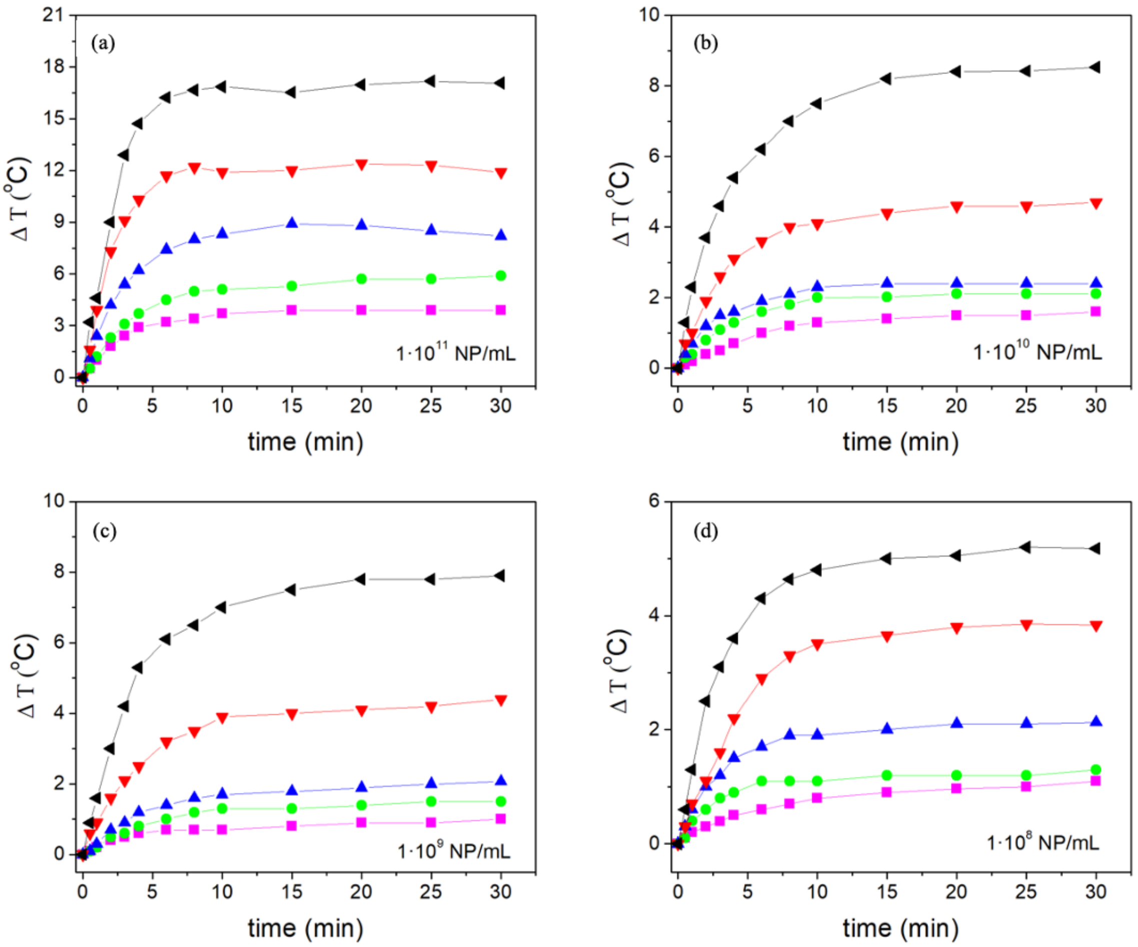

Figure S3: Temperature increases (ΔT) for AuNRs having a longitudinal LSPR band centered at 816 nm as a function of time under NIR light irradiation (808 nm) of intensities (■) 0.25, (●) 0.5, (▲) 1.0, (▼) 2.0 and (◀) 3.0 W/cm² for particle concentrations of (a) $1{\cdot}10^{11}$, (b) $1{\cdot}10^{10}$, (c) $1{\cdot}10^{9}$, (d) $1{\cdot}10^{8}$ AuNRs/mL. Lines are only to guide the eye.

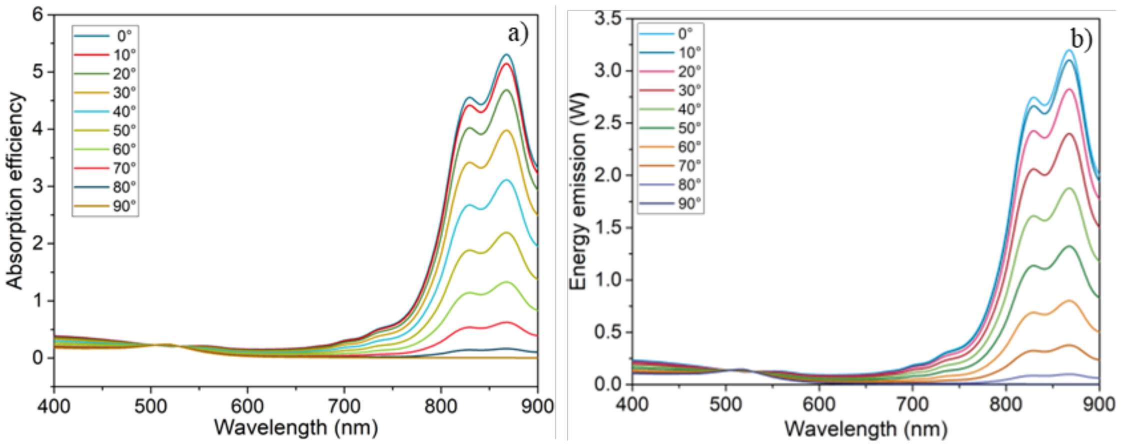

Figure S4: (a) Absorption efficiency (calculated as the ratio of absorption cross section and geometric cross section), and (b) energy emission under laser light irradiation of 1.0 W/cm² (808 nm) for 5 min under different polarization angles.

Influence of ionic strength on the LbL coating of AuNRs

We investigated the influence of salt concentration and buffer composition on polyelectrolyte (PE) adsorption onto AuNRs to provide them with sufficient colloidal stability, biocompatibility and cargo loading capabilities. Particle coating was performed in the presence of NaCl concentrations ranging from 0 to 12 mM at pH 4.1. ζ-potential and UV-vis measurements allowed to follow the influence of ionic strength on the physico-chemical properties of the polymer-coated AuNRs (Figure S5a-b). A blue shift of 8 nm in the LSPR band of the NPs could be noted regardless the ionic strength as consequence of changes in the refractive index of the NP surrounding medium due to the polymer wrapping around the particle surfaces. ζ-potential values continuously decreased until 3 mM NaCl to subsequently leveling off. This pointed that starting from this ionic strength, the PSS chains are flexible enough to successfully wrap around AuNRs, with a great number of polymer chains coating the NP. Moreover, it is worth mentioning that neither flocculation nor particle aggregation were observed at any analysed salt concentration.

On the other hand, once determined the suitable ionic strength for PE coating, DOXO incorporation was optimized by slightly changing the solution composition at pH 4.1. At this respect, an acetic acid buffer at this pH, a water solution with KOH equilibrated with HCl to reach pH 4.1 and an acetic acid/KOH buffer also at pH 4.1 were used. In previous papers, only maximum fed initial DOXO amounts of up to 300 µg were possible to be used without compromising the stability of the NPs, reaching an encapsulation efficiency (EE%) for such initial loading of ca. 25%. In the present case, it was observed that the presence of KOH at acidic condition (pH 4.1) favors the initial stability of the platform and allow to incorporate up to 350 µg, with an EE% of ca. 70%. For CH_3COOH or a CH_3COOH+KOH buffers at pH 4.1 the drug loadings were ca. 298 and 309 µg, with EE% of 60 and 62%, respectively, but still much higher than in previous reports [S1]. Moreover, the aqueous solution+KOH at pH 4.1 perfectly maintains the stability of the encapsulated drug within the nanoplatform as denoted by the constancy of its surface charge and optical drug absorbance at ca. 480-485 nm upon time; conversely, important optical and electrical changes are noted under the other solution conditions tested (Figure S5c-h).

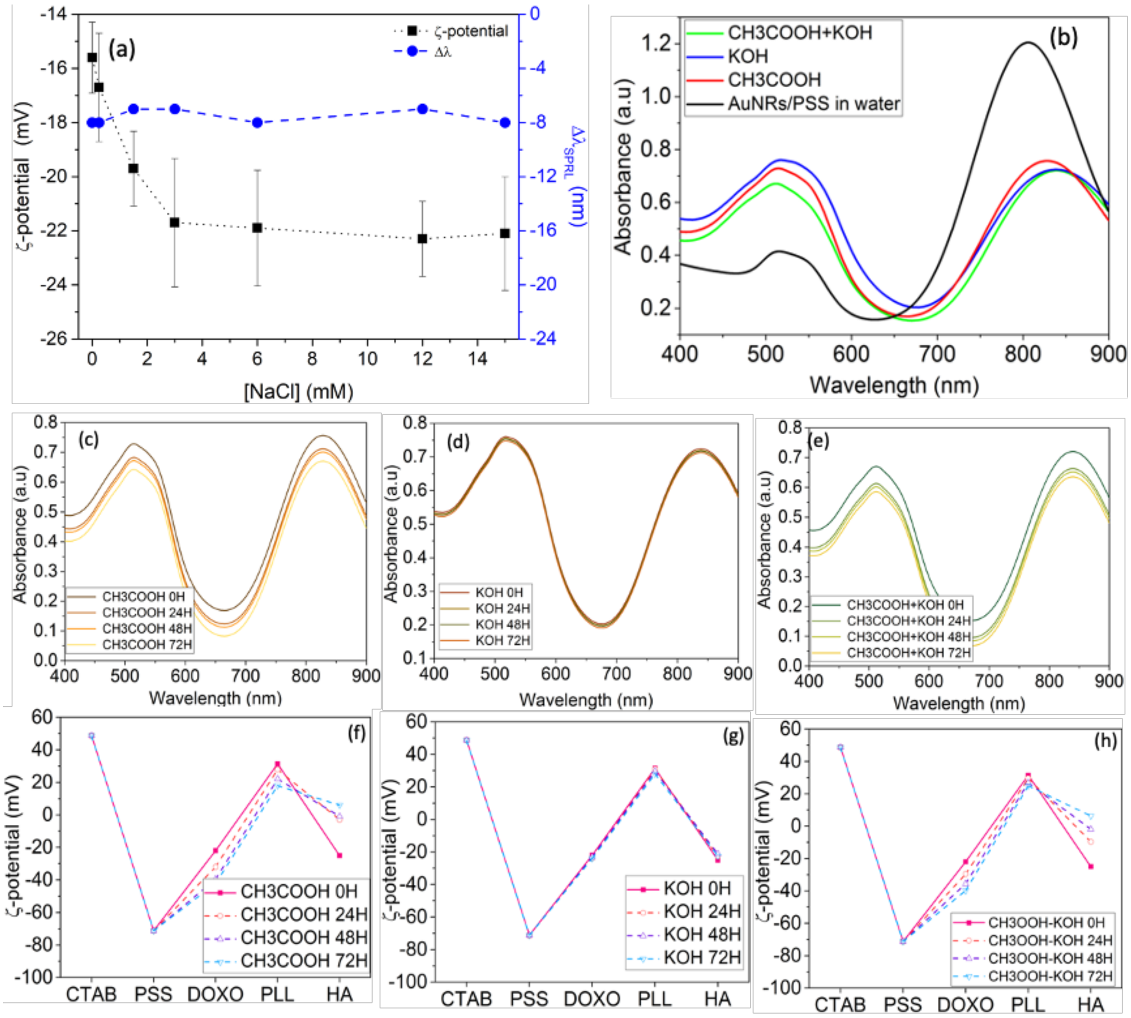

Figure S5: (a) ζ-potentials (■) and LSPR shifts (●) of PSS-coated AuNRs at different NaCl concentrations. Dotted and dashed lines are only to guide the eye. (b) UV-Vis spectra of PSS/DOXO/PLL/HA-coated AuNRs at different solution conditions for drug encapsulation/coating. Time stability of DOXO coating in PSS/DOXO/PLL/HA-coated AuNRs at different drug-loading solution conditions and incubation times as determined by (c-e) UV-Vis and (f-h) ζ-potential data.

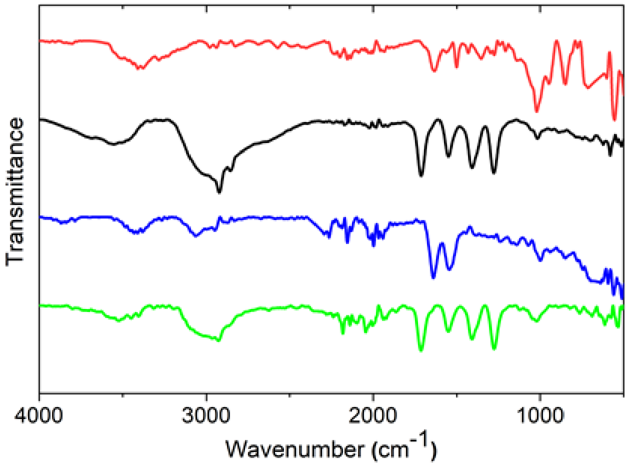

Figure S6: FTIR spectra of (—) free DOXO; and AuNRs coated with (—) CTAB; (—) PSS/PLL and (—) PSS/DOXO/PLL/HA.

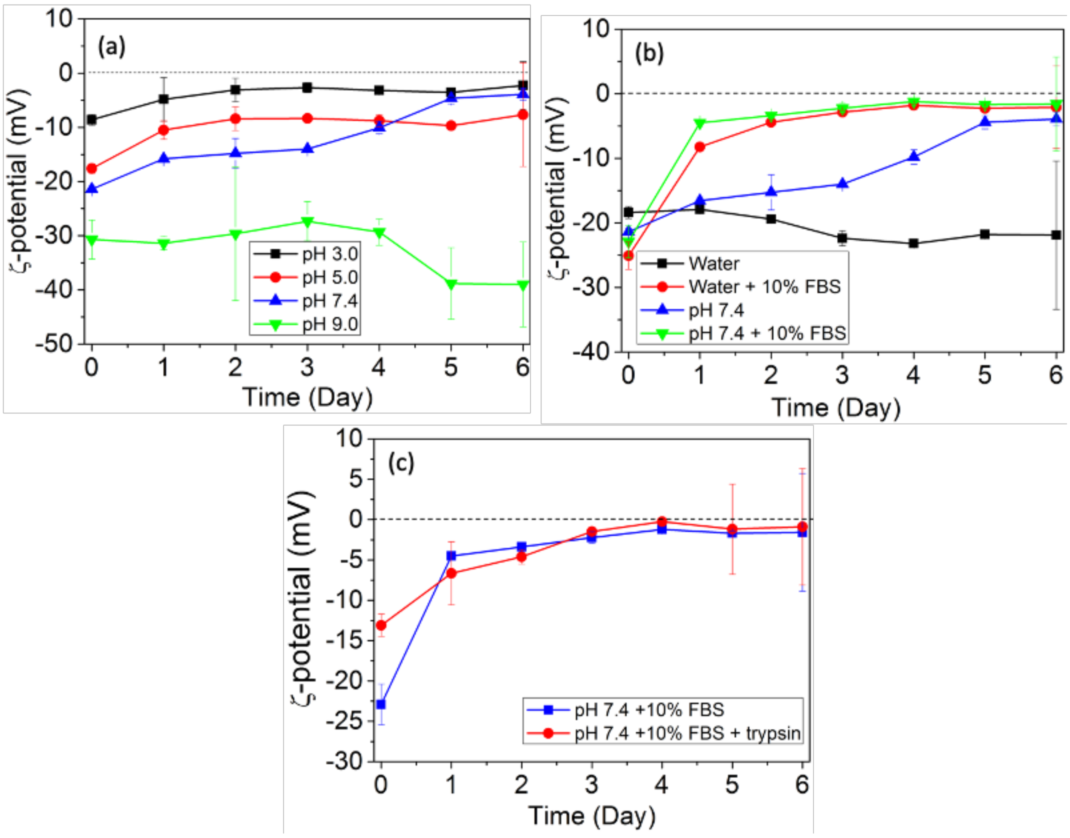

Figure S7: Colloidal stability of PSS/PLL/HA-coated AuNRs by monitorization of the time evolution of their surface charge at (a) different pH, (b) in the absence and presence of serum, and (c) in the absence and presence of trypsin at 37 ºC. Samples were diluted in buffers of pH 3.0 (■), 5.0 (●), 7.4 (▲), and 9.0 (▼).

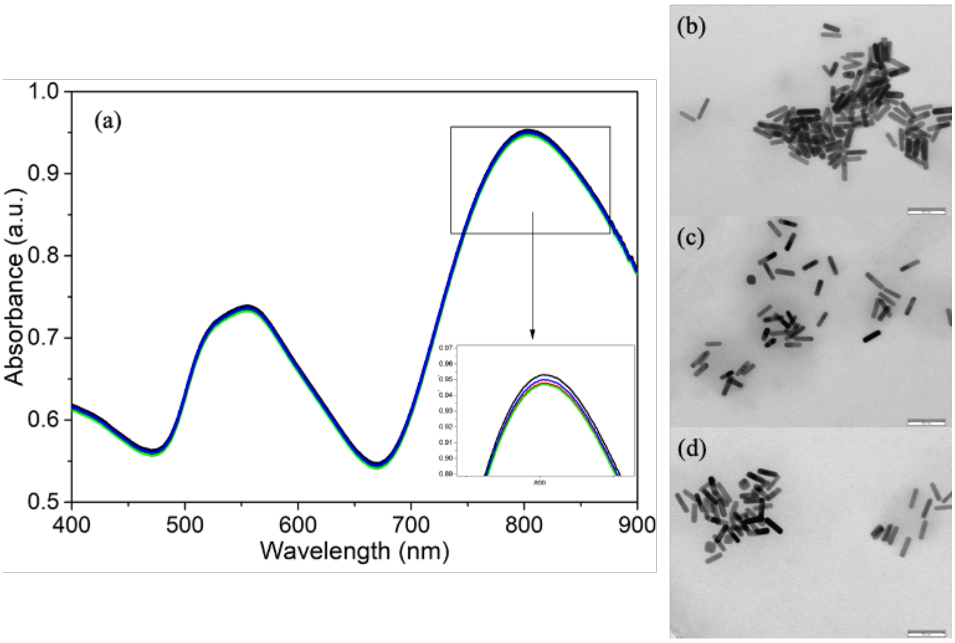

Figure S8: (a) UV-Vis spectra of PSS/PLL/HA-coated AuNRs after (—) 0, (—) 1, (—) 2, and (—) 3 irradiation cycles at a fluency of 2.0 W/cm². TEM images of PSS/PLL/HA-coated AuNRs after (b) 1, (c) 2, and (d) 3 irradiation cycles. Scale bar is 50 nm.

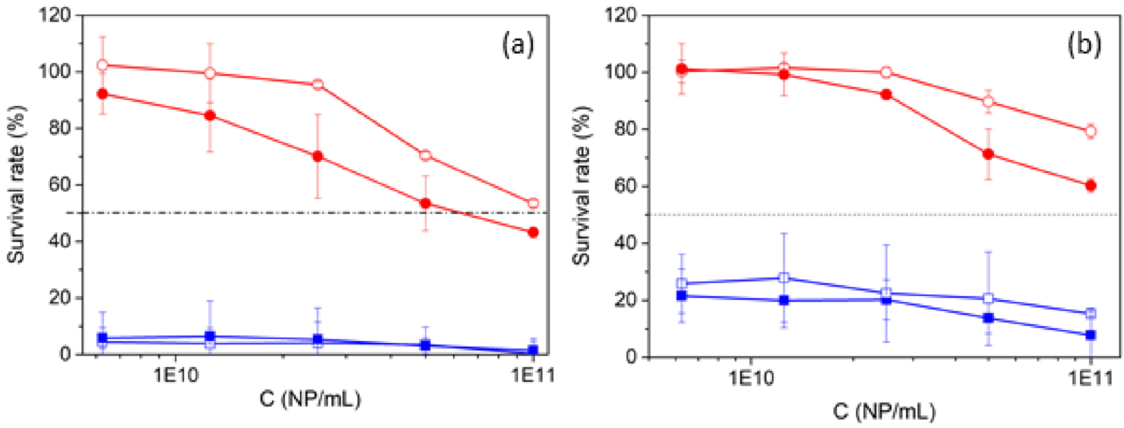

Figure S9: Cellular toxicity expressed as survival rate for (■, □) CTAB-, and (●, ○) PSS/PLL/HA-coated AuNRs, (a) tumoral cervival HeLa cells and (b) 3T3-Balb fibroblasts after 24 (open symbols) and 48 h (close symbols) of incubation, respectively.

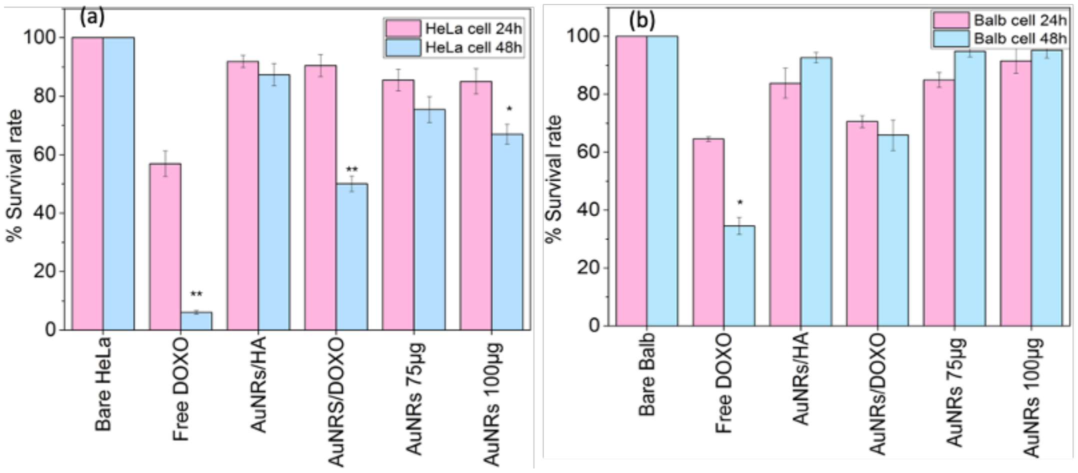

Figure S10: Cellular cytotoxicity expressed as survival rate of (a) HeLa and (b) 3T3 Balb cells at different incubation times at 37 ºC in the absence of NIR light illumination. AuNRs/HA denote PSS/PLL/HA-coated NPs, AuNRs/DOXO correspond to PSS/DOXO-coated AuNRs, and AuNRs 75μg and AuNRs 100μg stand for PSS/DOXO-PLL/HA-coated AuNRs encapsulating 75 and 100 μg of the drug, respectively. Statistical significance compared to 4ºC at each group: * = $P < 0.05$; ** = $P < 0.01$.

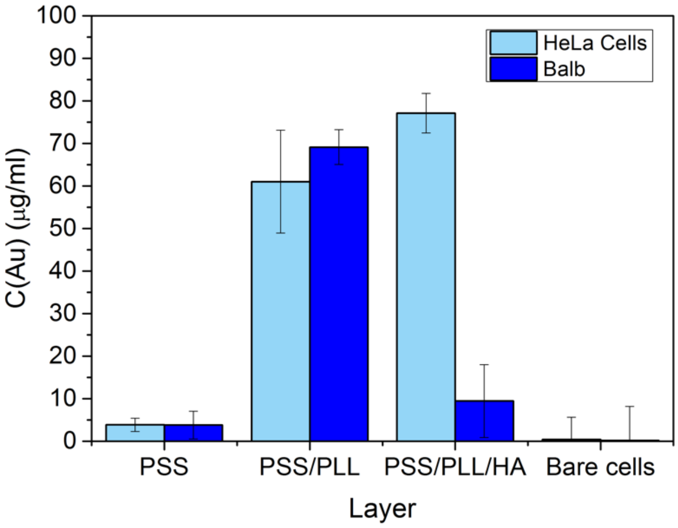

Figure S11: Quantification of cellular uptake of the nanoplatform at different stages of LbL polymeric deposition in HeLa and 3T3 Balb cells by means of ICP-MS.

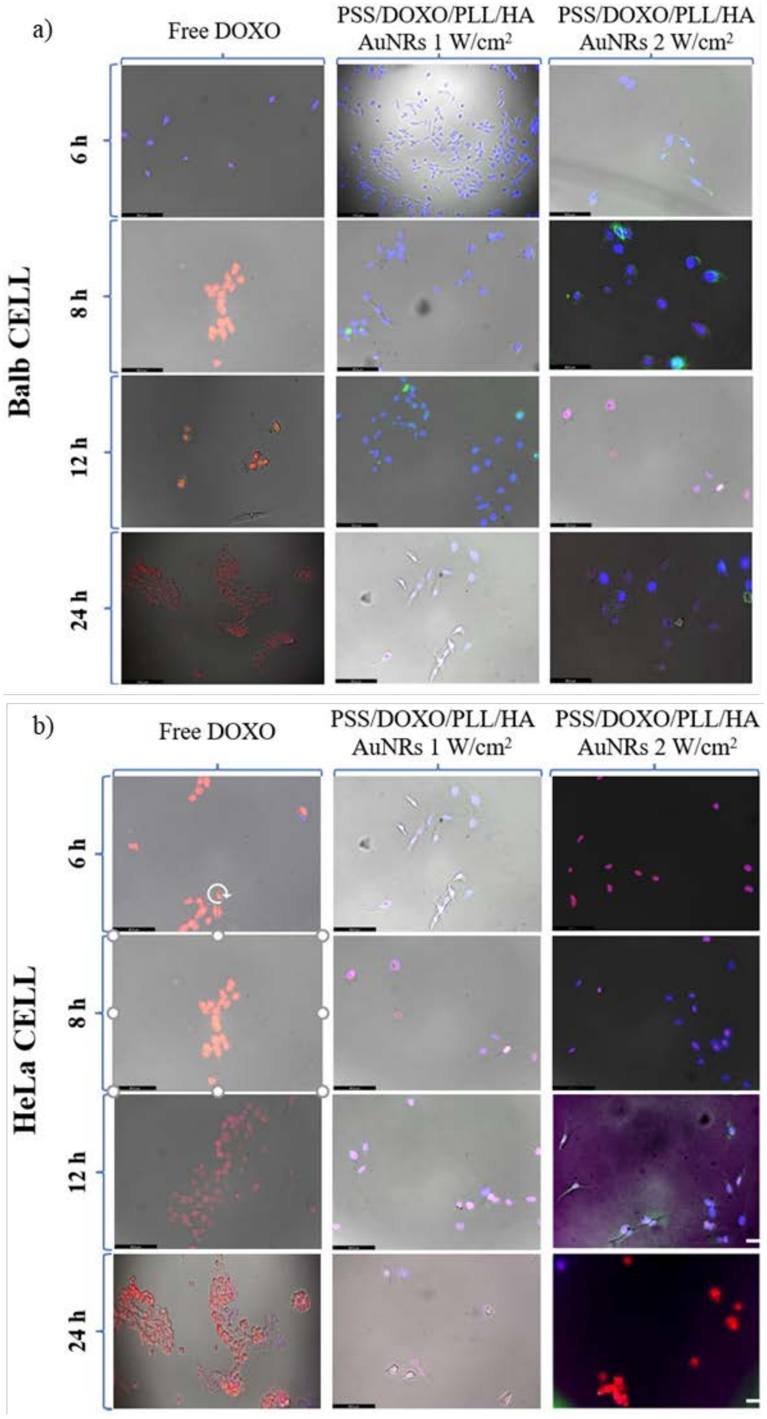

Figure S12: Merged fluorescence microscopy images of free DOXO and PSS/DOXO/PLL/HA-coated AuNRs inside (a) 3T3 Balb and (b) HeLa cells at different incubation times and NIR irradiation conditions. Blue corresponds to cell nuclei stained with DAPI (λ_{ex} = 355 nm) and red to DOXO fluorescence (λ_{ex} = 488 nm). Scale bars are 10 µm.

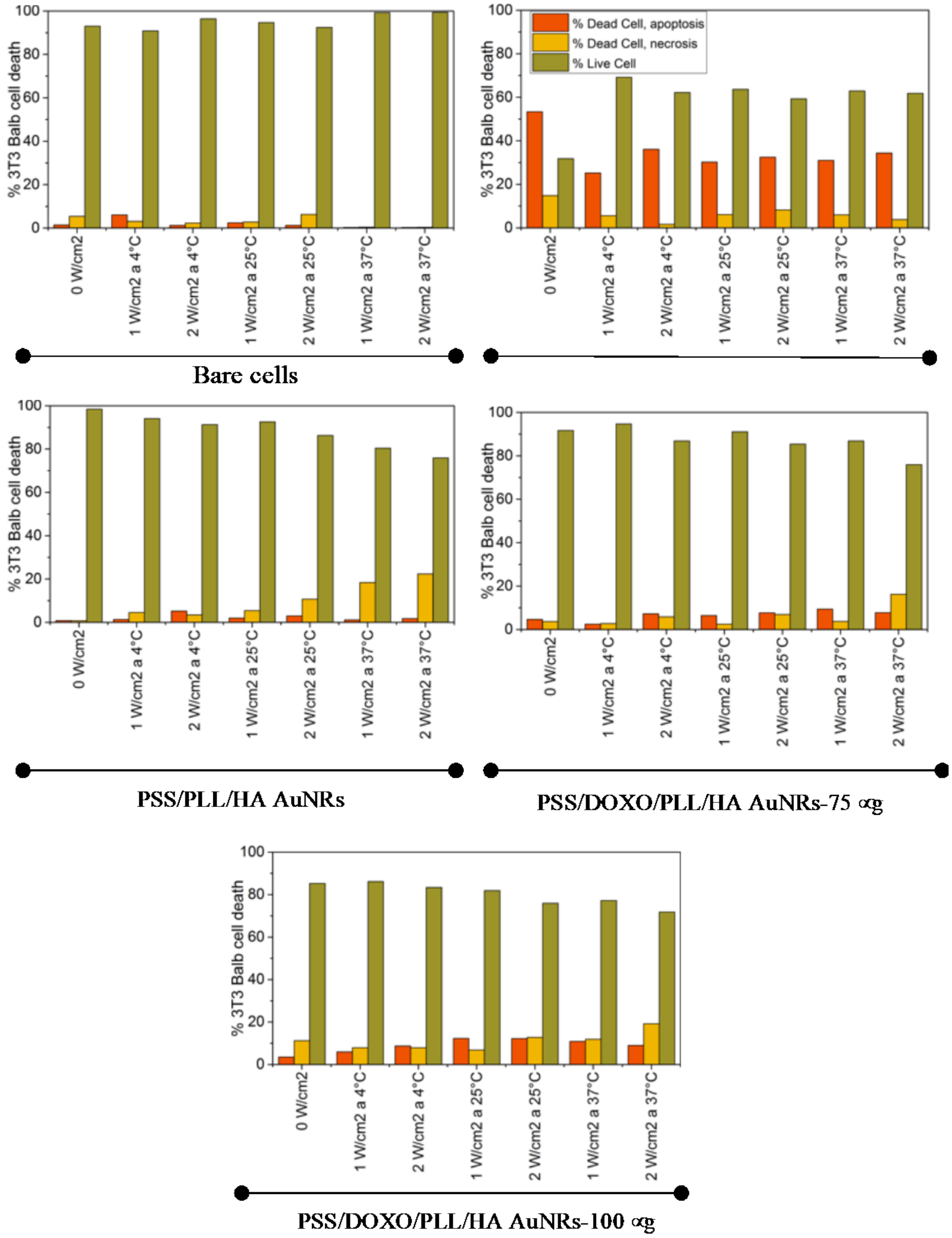

Figure S13: Main cell death mechanisms involved in cell mortality after administration of free DOXO, bare PSS/PLL/HA-coated and PSS/DOXO/PLL/HA-coated AuNRs with 75 and 100 μg DOXO for 3T3 Balb cells and at different temperatures and NIR fluencies.

References

[S1] Villar-Alvarez, E., et al., *Gold Nanorod-Based Nanohybrids for Combinatorial Therapeutics.* ACS Omega, 2018. 3(10): p. 12633-12647.
